# Carbon Flux as a Measure of Prostate Cancer Aggressiveness: [^11^C]-Acetate PET/CT

**DOI:** 10.7150/ijms.39542

**Published:** 2020-01-14

**Authors:** Naresh Regula, Hadis Honarvar, Mark Lubberink, Håkan Jorulf, Sam Ladjevardi, Michael Häggman, Gunnar Antoni, Jos Buijs, Irina Velikyan, Jens Sörensen

**Affiliations:** 1 Division of Nuclear Medicine and PET, Department of Surgical Sciences, Uppsala University, Uppsala, Sweden; 2 Division of Urology, Department of Surgical Sciences, Uppsala University, Uppsala, Sweden; 3 Division of Molecular Imaging, Department of Surgical Sciences, Uppsala University, Uppsala, Sweden; 4 Medical Physics, Uppsala University Hospital, Uppsala, Sweden; 5 Department of Immunology, Genetics and Pathology, Uppsala University, Uppsala, Sweden; 6 PET Centre, Uppsala University Hospital, Uppsala, Sweden

**Keywords:** carbon-11 acetate, positron emission tomography, prostate cancer, dynamic imaging

## Abstract

**Purpose**: Dynamic [^11^C]-acetate positron emission tomography (PET) can be used to study tissue perfusion and carbon flux simultaneously. In this study, the feasibility of the quantification of prostate cancer aggressiveness using parametric methods assessing [^11^C]-acetate kinetics was investigated in prostate cancer subjects. The underlying uptake mechanism correlated with [^11^C]-acetate influx and efflux measured in real-time in vitro in cell culture.

**Methods**: Twenty-one patients with newly diagnosed low-to-moderate risk prostate cancer underwent magnetic resonance imaging (MRI) and dynamic [^11^C]-acetate PET/CT examinations of the pelvis. Parametric images of K_1_ (extraction × perfusion), k_2_ (oxidative metabolism) and V_T_ (=K_1_/k_2_, anabolic metabolism defined as carbon retention) were constructed using a one-tissue compartment model with an arterial input function derived from pelvic arteries. Regions of interest (ROIs) of the largest cancer lesion in each patient and normal prostate tissue were drawn using information from MRI (T2 and DWI images), biopsy results, and post-surgical histopathology of whole prostate sections (n=7). In vitro kinetics of [^11^C]-acetate were studied on DU145 and PC3 cell lines using LigandTracer^®^ White equipment for the measurement of the radioactivity uptake in real-time at 37°C.

**Results**: Mean prostate specific antigen (PSA) was 8.33±3.92 ng/mL and median Gleason Sum 6 (range 5-7). K_1_, V_T_ and standardized uptake values (SUVs) were significantly higher in cancerous prostate tissues compared to normal ones for all patients (p<0.001), while k_2_ was not (p=0.26). PSA values correlated to early SUVs (r=0.50, p=0.02) and K_1_ (r=0.48, p=0.03). Early and late SUVs correlated to V_T_ (r>0.76, p<0.001) and K_1_ (r>0.64, p<0.005). In vitro studies demonstrated higher extraction and retention (p<0.01) of [^11^C]-acetate in the more aggressive PC3 cells.

**Conclusion**: Parametric images could be used to visualize the [^11^C]-acetate kinetics of the prostate cancer exhibiting elevated extraction associated with the cancer aggressiveness. The influx rate of [^11^C]-acetate studied in cell culture also showed dependence on the cancer aggressiveness associated with elevated lipogenesis. Dynamic [^11^C]-acetate/PET demonstrated potential for prostate cancer aggressiveness estimation using parametric-based K_1_ and V_T_ values.

## Introduction

Prostate cancer (PCa) is one of the most common malignancies in men with an estimated global number of new cases of 1,111,700 and an estimated mortality of 307,000 men per year 1. The typical methods for monitoring patients with assumed low-risk tumors are measurements of serum prostate specific antigen (PSA) level, repeated prostate biopsies, and magnetic resonance imaging (MRI) 2, 3. Patient stratification based solely on PSA level is controversial due to the high rate of overdiagnosis, and subsequent unnecessary prostate biopsy and surgery. MRI reduces the risk for the overdiagnosis and is the most robust morphological imaging modality with high predictive capacity in the management of PCa 4. However, the introduction of diagnostic imaging methods that would allow for quantification of the PCa progression on a molecular level could lead the patient management to the level of personalized medicine. It is of great importance especially in the case of indolent PCa.

Three main metabolic distinctive features in a cancerous cell are: avid glucose consumption (aerobic glycolysis); high energy consumption to drive increased protein and DNA synthesis; and an increased de novo fatty acid (FA) synthesis. In PCa, an increase in aerobic glycolysis is only found in advanced disease, whereas de novo FA synthesis and high energy consumption are common features of both primary and advanced PCa 5. Like any other cancer cell type, PCa cells show great demand of cell membrane lipids for growth and proliferation 6.

Selective targeting of distinguishing features of cancer cells is the new strategy in molecular cancer therapeutics. Fatty acid synthetase (FASN), the rate limiting enzyme in de novo FA synthesis, converting excess carbon intake into fatty acids for storage, seems to be an attractive potential target for new therapeutic approach 5. Though, several FASN inhibitors had showed antitumor activity, their mechanism is still not well understood. Moreover, large variations in FASN expression levels in individual tumors pose a challenge to predict therapeutic outcome. To avoid unnecessary treatment, methods to stratify those who gain benefit with the application of FASN targeted therapy are needed.

One approach to this requirement is focusing on the quantification of the uptake of radiolabeled acetate ([^11^C]-acetate) using positron emission tomography (PET). Acetate, as an important intermediary substrate, is the main carbon source for fatty acid and lipid synthesis 7. The intracellular fate of acetate is diverse. To use acetate, cells must activate it into acetyl-CoA which is regulated by acetyl-CoA synthetase (AceCS). Two distinctive forms of mammalian AceCS enzymes are identified and are localized over mitochondria and cytosol 8. Acetate entering the mitochondria, under the regulation of mitochondrial AceCS and other enzymes, is metabolized to CO_2_ and leaves the cell reflecting the oxidative metabolism. This pathway has been exploited with [^11^C]-acetate PET combined with computed tomography (CT) to quantify the myocardial oxidative metabolism 9-14. Whereas cancerous cells govern the use of acetate for de novo lipogenesis wherein FASN is the rate limiting enzyme.

Previous studies showed that an increase in FA synthesis seems to be an early event in tumorigenesis and is correlated with the progression of the disease. Preclinical in vitro and in vivo studies have demonstrated a correlation of [^11^C]-acetate uptake with enhanced FASN 15-17 and cytosolic AceCS 18 expression. Moreover, a clinical study in 123 consecutive patients showed that FASN was upregulated in PCa and its expression was correlated to tumor aggressiveness in terms of [^11^C]-acetate accumulation 19. These studies proved [^11^C]-acetate PET/CT to be a promising metabolic tracer for primary staging and for localizing recurrent disease. Moreover, [^11^C]-acetate PET/CT was found superior to ^18^F-FDG PET/CT in terms of sensitivity 20 and identical to ^18^F-choline PET/CT in terms of detection rate and uptake intensity 21. Regula et al. have shown that the standardized uptake value (SUV) along with PET volumetrics of [^11^C]-acetate PET/CT serve as predictors of survival in biochemical recurrent PCa patients after surgery 22. However, SUV does not explain the underlying tumor biology completely, and the preclinical studies linking radiolabeled acetate uptake with FASN expression failed to show the dynamic changes occurring with the consumed acetate at the cell level. Contrary to SUV, parametric images of [^11^C]-acetate kinetics based on dynamic PET data can show perfusion and oxidative metabolism at the voxel level.

The significance of perfusion and tumor oxidative metabolism in cancer growth inhibition was demonstrated clinically proving the potential of non-invasive [^11^C]-acetate PET/CT imaging for the investigation of bioenergetic mechanisms of prostate cancer 23. Yoshii et al. showed in their experimental work that inhibition of FASN reduces the tumor activity in terms of [^11^C]-acetate uptake 18. More fundamental understanding of the role of perfusion, oxidative and anabolic metabolism of PCa would potentially improve the accuracy of tumor diagnosis and prediction of treatment outcome. The investigation of [^11^C]-acetate uptake mechanism and role of perfusion and metabolism can be aided by kinetic modelling based on dynamic scanning of the patients. In particular, the overall [^11^C]-acetate uptake could be well described by a 3-compartment model 24, however fundamental studies of separate aspects of the uptake process require further studies employing respective kinetic models and clear correlation of the results with in vitro experiments designed to reveal the mechanism of the particular aspects.

The aim of this study was, therefore, to investigate the tumor biology using single-tissue compartment model derived parametric images of [^11^C]-acetate dynamic PET/CT and to correlate the results with [^11^C]-acetate dynamic data from cell experiments revealing the metabolic activity in real-time.

## Materials and Methods

### Dynamic [^11^C]-acetate Clinical setting

#### Patients

Twenty-one patients with newly diagnosed and biopsy proven low-to-moderate risk prostate cancer were included in this prospective study and were scanned between the end of 2009 and the beginning of 2011. Mean age was 65 y (range, 51-75 y). PSA at the time of PET was 8.33±3.92 ng/mL. Median Gleason score of the subjects was 6 (range, 5-7). Pelvic magnetic resonance imaging (MRI) with different sequences such as T2-weighted and diffusion-weighted was performed in all patients prior to dynamic PET/CT. Seven of 21 patients underwent surgery and the resected prostate gland was sent for histopathology.

Ethical approval for this prospective study was obtained from the Regional Ethics Committee (EPN 2009/191) and all patients signed informed consent before inclusion.

#### PET/CT protocol

[^11^C]-acetate was synthesized according to the [^11^C]-acetate simple synthesis method proposed by Le Bars et al. 25 based on original synthesis 26 with in-house modifications. After a 6 h fast to avoid any variation in the measurement due to the influence of food intake, all patients underwent a dynamic PET scan starting simultaneously with intravenous injection of 5 MBq/kg [^11^C]-acetate. PET was performed using a GE Discovery ST16 scanner (GE Healthcare, Waukesha, WI) with a spatial resolution of 5 mm at the center of the field of view. Thirty-two frames were acquired of 12×5s, 6×10s, 4×30s, 4×60s, 2×120s, and 4×300s duration. Prior to PET, a CT transmission scan (140 kV, 40-80 mA) without contrast medium was obtained.

PET images were reconstructed using ordered subsets expectation maximization (OSEM) using 2 iterations and 21 subsets, applying all appropriate corrections.

#### Image processing

PET/CT images were analysed using VOIager (GE Healthcare, Uppsala, Sweden). Volume of interests (VOIs) were drawn over iliac vessels and time activity curves (TAC) were extracted which served as an arterial input function for the kinetic model. Early (2-5 min) and late (22-32 min) standardized uptake value (SUV) images were derived from dynamic PET and analysed.

#### Kinetic Model

[^11^C]-acetate kinetics were modelled using a single-tissue compartment model, which explains how [^11^C]-acetate is transported across the cell membrane and a fraction undergoes oxidative metabolism by entering the Krebs cycle, while the remaining is added to the intracellular metabolic pool and used further in anabolic metabolism.

**Figure 1:**
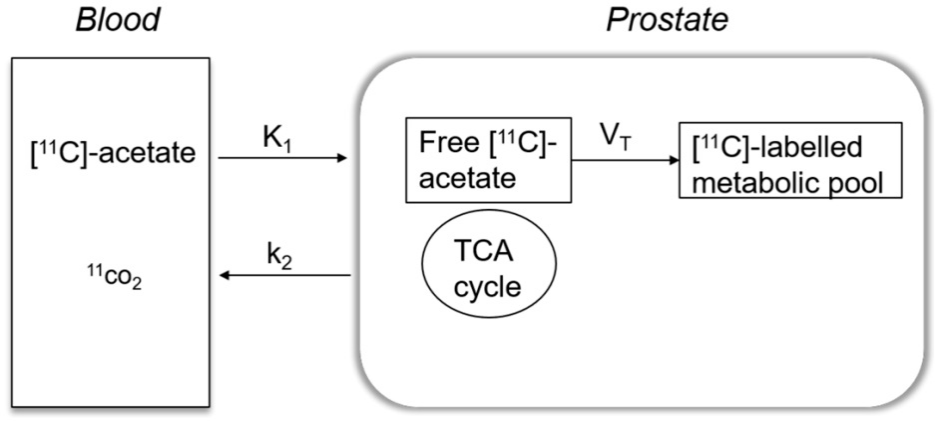
Simple one tissue compartment model describing the kinetics of [^11^C]-acetate in a prostate cancer cell. K_1_ reflects the extraction rate of [^11^C]-acetate into prostate cell and k_2_ indicates the fraction that undergoes oxidative metabolism in mitochondria and leaves the cell as ^11^CO_2_. The remaining [^11^C]-acetate is distributed (V_T_) within the prostate cell used for anabolic metabolism. TCA: mitochondrial Krebs cycle.

A basis-function implementation of the single-tissue compartment model as shown in Figure 1 yielded parametric images of K_1_, k_2_ and V_T_. K_1_ indicates the transport rate of [^11^C]-acetate into prostate which is the product of extraction and perfusion (mL/cm^3^/min) and k_2_ denotes the rate of oxidative metabolism (min^-1^). V_T_ reflects the amount of [^11^C]-acetate volume distributed within prostate tissue used for anabolic metabolism (mL/cm^3^). Correction for the metabolic stability of [^11^C]-acetate was applied as previously described 24. A 6-mm filter was applied for smoothing of the k_2_ parametric image.

From the histopathology report (n=7) and based on MRI data, the cancerous region in prostate was differentiated from normal tissue and separate region of interests (ROIs) were drawn over these areas on parametric images for the subsequent analysis.

### In vitro dynamic [^11^C]-acetate

The human prostate cancer cell lines DU145 and PC3 (ATCC, Germany) were used in the cell experiments. Cells were cultured in RPMI medium supplemented with 10% fetal calf serum, 2 mM L-glutamine and PEST (penicillin 100 IU/mL and 100 μg/mL streptomycin) (Biochrom AG). DU145 and PC3 cells were seeded on a local area of a cell culture dish. The cells were incubated overnight, and they formed a confluent layer of cells by the time of the experiment (NunclonTM, Size 100620, NUNC A/S, Roskilde, Denmark). The uptake and retention of [^11^C]-acetate in living cells was monitored in real-time at 37°C using LigandTracer^®^ White (Ridgeview Instruments AB, Uppsala, Sweden) placed in an incubator 27, 28. The cell culture dish was placed on an inclined rotating support. The radioactivity detection unit was above the upper part of the dish and the inclination ensured that the 3 mL [^11^C]-acetate containing medium was mainly outside the detection area. During each full rotation, the signal from the cell and a reference area was recorded. The reference signal was automatically subtracted and corrected for [^11^C] decay resulting in a real-time signal that represented cell associated [^11^C]-acetate both during incubation and after the wash. For experiments where the acetate uptake was linear, the relative acetate uptake rate was quantified by dividing the uptake rate, i.e. the decay corrected signal increase per min, by the average signal derived from the reference area during incubation which provided a relative measure of the amount of [^11^C]-acetate added to the dish.

### In vitro kinetics evaluation of [^11^C]-acetate

In a kinetic assay, the [^11^C]-acetate uptake and retention rates by PC3 and DU145 cell lines were evaluated under the same conditions and the experiments were performed in parallel using two LigandTracer^®^ devices. The uptake of [^11^C]-acetate by DU145 and PC3 cells was measured for ca. 15 min after adding 500-2000 kBq of the radiotracer. In a set of experiments, the uptake of [^11^C]-acetate was followed up for 120 min after incubation. Thereafter, the tracer containing solution was replaced with fresh medium, after washing the dish two times with medium. The retention of [^11^C]-acetate in the cells was followed for around 30-45 min.

To check the uptake saturability of [^11^C]-acetate, two assays were performed where the uptake was monitored over time using LigandTracer^®^. In a titration assay, cells were incubated with increasing concentrations of [^11^C]-acetate, selected based on the amount of activity, i.e. 200, 600, 1800, 3000 kBq.

In a competition assay, two experiments were performed in parallel on PC3 cells. In both experiments about 1MBq of [^11^C]-acetate was added to PC3 cells and the uptake of [^11^C]-acetate was monitored for 15min. Thereafter, 100 mmoles of acetic acid was added to one of the dishes and the uptake of [^11^C]-acetate by PC3 cells was monitored further during up to 100 min.

### In vitro [^11^C]-acetate uptake per cell

A study was performed in which DU145 and PC3 cells were seeded in 6 petri dishes, i.e., three sets of dishes per cell line. About 3MBq [^11^C]-acetate was added to each dish and incubated at 37℃ for 15 min. Thereafter, the radioactive solution was removed, and the cells were washed twice with ice-cold serum free medium. Then, the cells were detached by trypsin-EDTA solution (0.25% trypsin, 0.02% EDTA in buffer, Biochrom AG, Berlin, Germany). The number of cells were counted, and the amount of the radioactivity was measured using a well-type NaI (Tl) scintillation counter, applying correction for dead-time and decay.

#### Statistical analysis

Data are presented as mean ±SD, unless otherwise stated. Differences between groups were evaluated using Wilcoxon signed-rank test. Correlation studies were conducted using Spearman's rank correlation coefficients. A two-tailed p-value of <0.05 was considered statistically significant. Statistical analyses were performed using JMP V12 (SAS Institute Inc., Cary, NC), unless otherwise stated.

## Results

### [^11^C]-acetate dynamic PET in humans

The clinical characteristics of the study population are shown in Table 1. The mean age of the study population was 65 years (range 51-75 years) with mean PSA level of 4.33±3.92 ng/mL and median Gleason of 6 (range 5-7).

#### [^11^C]-acetate uptake pattern

**Figure 2:**
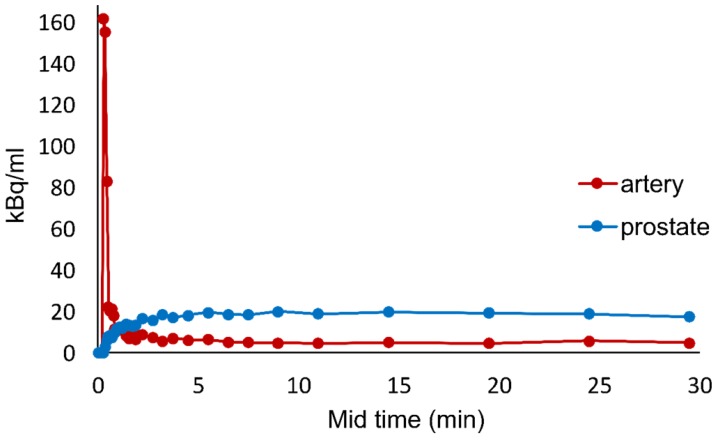
Typical example of time activity curve generated for one of the patients from VOI placed over iliac vessels and prostate tissue.

A typical example of the time activity curve is shown in Figure 2. The lesion tissue curve has an initial incline and reaches a plateau at around 5 mins. Analysis of time activity curves revealed that early uptake (early SUV), from 2 to 5 minutes on average was similar to the late uptake from 22 to 32 minutes (late SUV).

**Figure 3:**
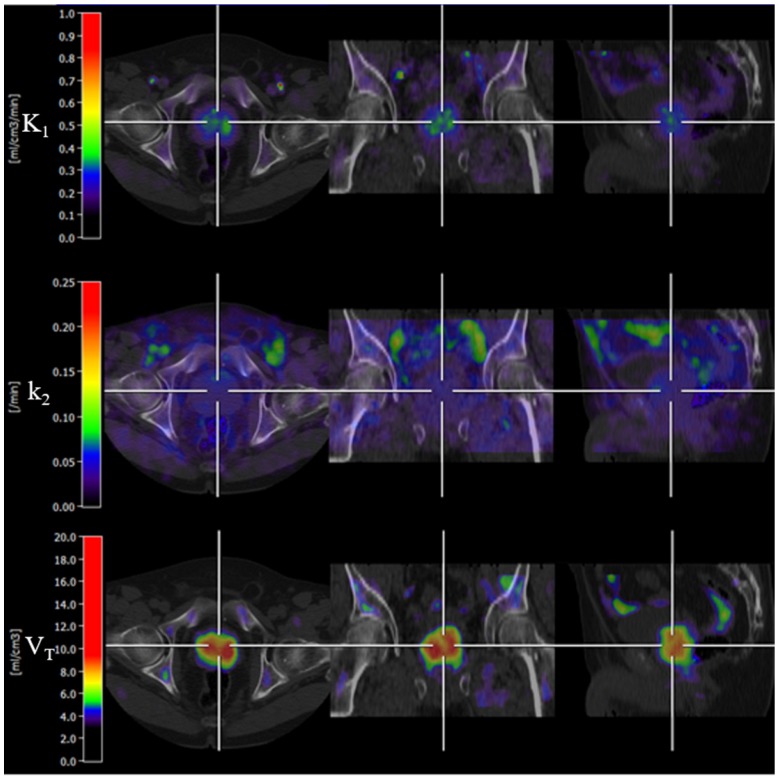
An example of parametric images of K_1_, k_2_ and V_T_ (Patient 2). V_T_ parametric image showed bilateral signal uptake in the prostate gland. The first, second and third rows demonstrate trans-axial, coronal and sagittal PET/CT fused images, respectively.

The basis function implementation of the single-tissue compartment kinetic model successfully produced parametric images in all subjects. An example of parametric images is shown in Figure 3. K_1_ and V_T_ of cancerous prostate on parametric images showed a significant difference compared with normal prostate.

The parametric values and the SUV from the dynamic scans of both normal and cancerous prostate are summarised in Table 2. No significant difference in oxidative metabolism (k_2_) was noted among different prostate regions.

A Spearman correlation test showed significant correlation of K_1_ and V_T_ with both early and late SUV and values are shown in Table 3. Bivariate analysis showed correlation of PSA to early SUV (r=0.50, p=0.02) and K_1_ (r=0.48, p=0.03).

### In vitro kinetics evaluation of [^11^C]-acetate

**Figure 4:**
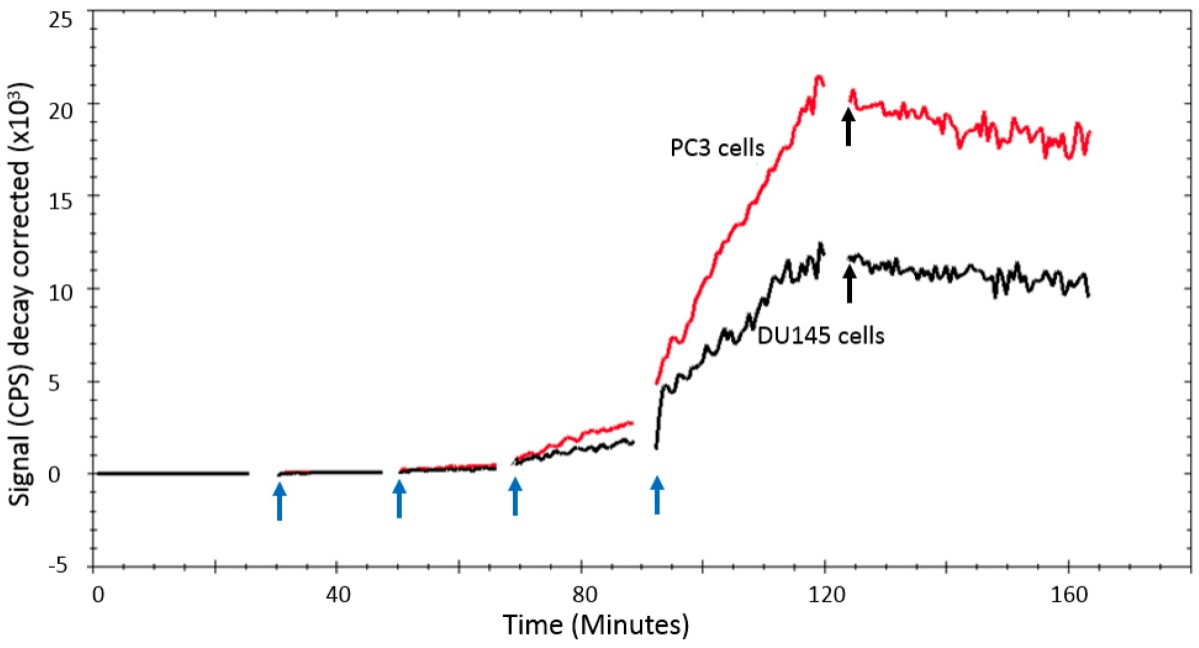
LigandTracer^®^ curve of [^11^C]-acetate uptake and retention on PC3 and DU145 cells. The [^11^C]-acetate concentration was gradually increased by subsequently adding 200, 600, 1800, and 3000 kBq (indicated by the blue arrows), respectively. [^11^C]-acetate retention was monitored after replacement of the incubation solution with medium after 125 min (indicated by the black arrows).

In a titration assay, it could be seen that [^11^C]-acetate uptake by DU145 and PC3 cells in vitro was characterized by a steady uptake that was proportional to the acetate concentration (Figure 4).

With the [^11^C]-acetate concentrations and incubation times used in the kinetic experiments, [^11^C]-acetate uptake was linear and proportional to the [^11^C]-acetate concentration in solution. The retention of [^11^C]-acetate in the cells was at least 45 min as demonstrated by the insignificant decrease of the [^11^C]-acetate signal when [^11^C]-acetate incubation solution was substituted with fresh media solution (Figure 4). The relative uptake rate of [^11^C]-acetate (mean ±SD, n=5) was 0.72 ± 0.24 h^-1^ for DU145 cells and 2.94 ± 0.12 h^-1^ for PC3 cells (p=0.002).

**Figure 5:**
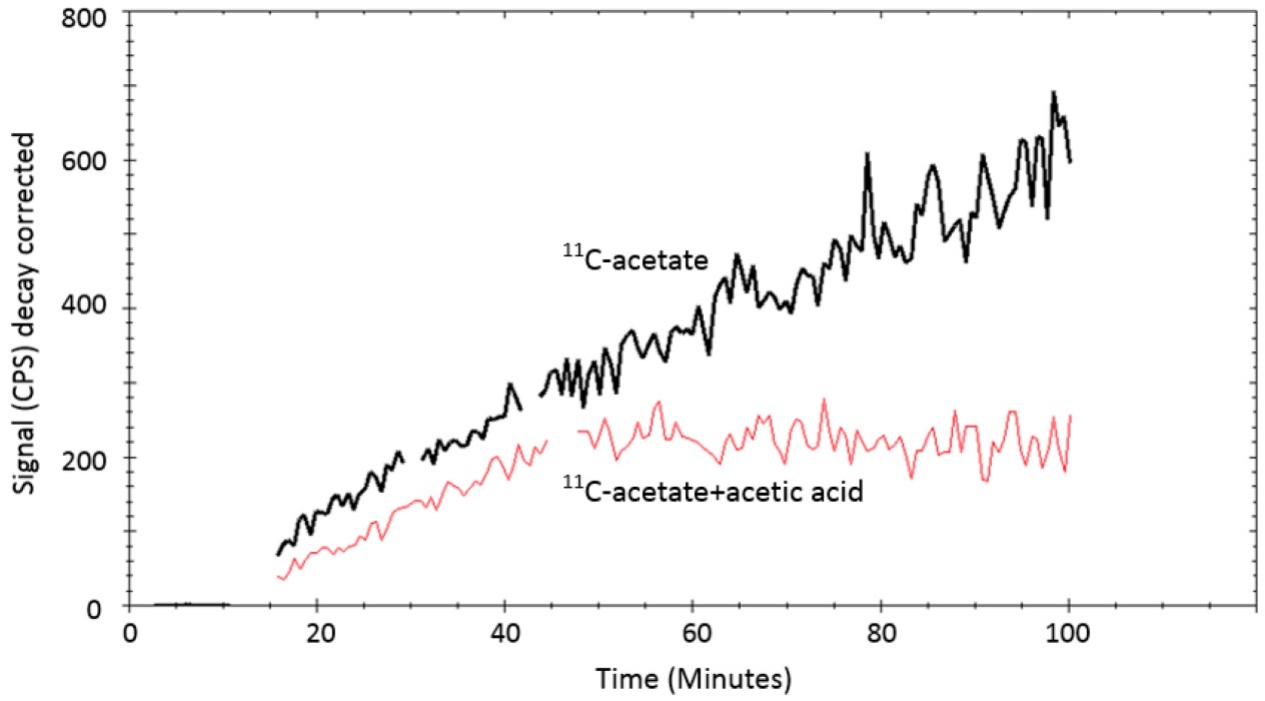
LigandTracer^®^ curves ([^11^C]-acetate: black curve, [^11^C]-acetate + acetic acid: red curve) of [^11^C]-acetate uptake on PC3 cells. The uptake of 1MBq [^11^C]-acetate was measured for 15min. Thereafter, 100 mmoles stable (non-labelled) acetic acid was added to one cell dish and the uptake of [^11^C]-acetate was measured further for up to 100 minutes. The uptake of [^11^C]-acetate reduced considerably in the presence of non-labelled acetic acid.

Excretion rates (mean ±SD, n=5) for DU145 and PC3 cells were 0.24± 0.06 h^-1^ and 0.52±0.04 h^-1^, respectively. The [^11^C]-acetate uptake was strongly reduced when excess of non-labeled acetate (100 mmoles) was added as seen in Figure 5.

### In vitro [^11^C]-acetate uptake per cell

**Figure 6:**
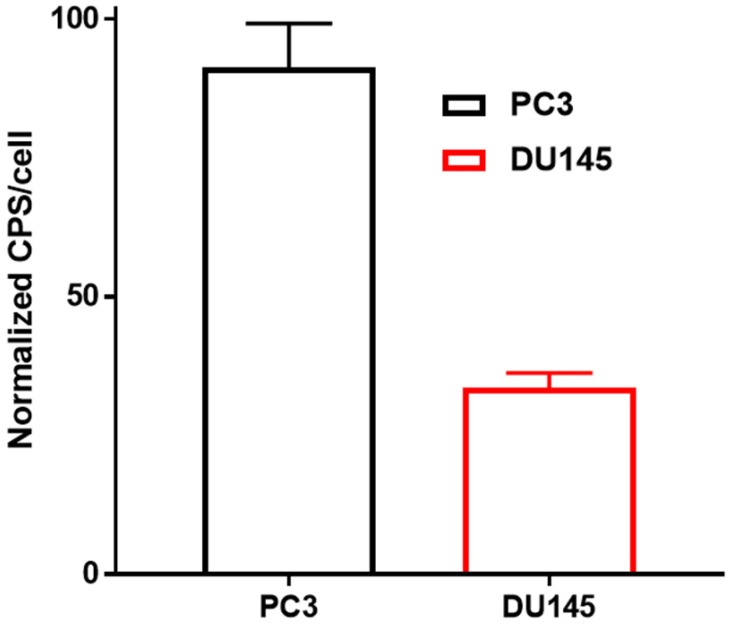
In vitro uptake of [^11^C]-acetate normalized to the amount of the cells: PC3 (black) and DU145 (red) 15 min after incubation at 37 °C. Results are presented as cps (count per second) per cell (cps/cell), error bars indicate 95% confidence interval of the mean. Statistically significant difference between cell lines was calculated according to Student's t-test resulting in a p-value of <0.001.

The uptake of [^11^C]-acetate by PC3 cells (cps/cell: 0.008±0.001) was about two times higher than that by DU145 cells (cps/cell: 0.003 ± 0.000) (Figure 6).

## Discussion

In this study, the kinetics of [^11^C]-acetate uptake in PCa subjects and prostate cancer cell lines were investigated. [^11^C]-acetate has previously been used for whole-body PET/CT imaging in PCa patients in primary staging and to localize the biochemical recurrent lesions in patients after primary treatment 20, 29-33. In this study, we applied a simple single-tissue compartment model to describe and visualize [^11^C]-acetate uptake kinetics with the aim to investigate the underlying uptake mechanism and respective tumor biology. This study demonstrated that the SUV of PCa measured in a human static whole-body image was primarily affected by initial delivery and only to a lesser extent by the clearance. This finding was confirmed by real-time kinetic studies of [^11^C]-acetate uptake by standard PCa cell clones in vitro.

### [^11^C]-acetate kinetics and prostate cancer biology

Localizing the cancerous region over the parametric images was aided by MRI and histopathology documentation. A single-tissue compartment model was successfully used to create parametric images of acetate kinetics (Figure 3). The input function was derived from the iliac vessels using VOI based method since the invasive arterial blood sampling was not feasible due to practical challenges. The metabolic stability correction was conducted to get the best fit between the measured and model derived time activity curves assuming that [^11^C]-labelled CO_2_ in tissue was negligible 24.

In the case of [^11^C]-acetate in tracer amounts, K_1_ equals the nutrient delivery rate and probably reflects perfusion, which is elevated in PCa in proportion to tumor aggressiveness 34, 35. Previously, K_1_ was calculated for primary, recurrent and benign lesions in PCa patients 24, however, comparison with control normal prostate tissue was not conducted. K_1_ in cancerous regions was determined using single- and two-tissue compartment kinetic models with or without correction for metabolic stability, and the resulting values varied ranging from 0.23 to 0.32 mL/cm^3^/min dependent on the number of included kinetic parameters 24. The simplified single-tissue compartment model used in our study resulted in similar value of 0.34 mL/cm^3^/min in cancerous prostate tissue. It was significantly higher than that in non-cancerous prostate (0.23 mL/cm^3^/min) and correlated with PSA values (r=0.48, p=0.03). The extracted K_1_ values may have been somewhat overestimated due to the incomplete recovery of the arterial blood curve and resulting partial volume effect, but this respective error was likely systematic and hence did not deteriorate the inter-patient comparison. The highest signal in [^11^C]-acetate K_1_ images correlated with the largest tumor nodule. This observation was confirmed in the cell line study wherein the more aggressive PC3 cells had higher uptake rate than DU145 cells, supporting the view that [^11^C]-acetate uptake is an indicator of PCa growth. Thus, [^11^C]-acetate was most probably taken up in proportion to fatty acid synthesis. In vitro kinetics property evaluation of [^11^C]-acetate showed that uptake of [^11^C]-acetate by DU145 and PC3 cells was increased continuously over time when the cells were incubated for up to 2h (data not shown). No saturation could be observed at any added concentration of [^11^C]-acetate (Figure 4) indicating the continuous consumption of the latter by the cells for lipogenesis. Moreover, the uptake rate of [^11^C]-acetate was significantly higher in faster growing PC3 cells than that for DU145. This observation is in concordance with previously published results demonstrating higher growth potential of PC3 cells as compared to that of DU145 cells 36. Thus, faster kinetics of [^11^C]-acetate presumably reflect the level of aggressiveness of the cancer cells. The [^11^C]-acetate uptake normalized to the cell amount (cps/cell) was almost three-fold higher for PC3 cells than that for DU145 (p<0.001) (Figure 6). In addition, the uptake of [^11^C]-acetate decreased substantially when 100 mmoles of acetic acid was added to the cells (Figure 5) indicating the displacement of [^11^C]-acetate by the excessive amount of acetic acid participating in the same specific biological process of lipogenesis.

Clearance rate (k_2_, Figure 3) of [^11^C]-acetate is used as an index for oxidative metabolism when applied in cardiac studies 11, 12, 37, 38, but oxidative metabolism in PCa is less well studied. There is evidence that forcing the cancer cell back into oxidative metabolism can inhibit cancer growth 39. Active oxidative metabolism in cancer might potentially be studied with dynamic [^11^C]-acetate PET. Using a similar technique as in the current study, Sun et al. found an indication that radiation therapy in head and neck cancers (H&N) with high oxidative metabolism resulted in more complete response 23. In H&N the clearance rate was calculated using simple mono-exponential fitting of the wash-out curve, resulting in a slight underestimation of the rates, compared to k_2_ from the kinetic modelling. Nonetheless, PCa appeared to have much higher rates of oxidative metabolism (0.04±0.01 min^-1^) in our study, compared to what was found in H&N (0.01±0.001 min^-1^). For comparison, mean k_2_ in normal left ventricular myocardium was 0.08±0.02 40. Post study follow-up showed that 14 out of 21 patients were primarily treated with radical prostatectomy, 1 had radiation therapy (RT) with curative intent, 4 were under active surveillance and 2 patients had no follow-up data. The data does not allow any conclusions on the association between oxidative metabolic rates and RT outcome. No significant difference of k_2_ between cancerous and normal regions of prostate was found in our study (p=0.26), suggesting that low-intermediate risk PCa and normal prostate depend on oxidative metabolism to the same extent.

The carbon retained within the cell and used for anabolic metabolism can be quantified by V_T_ which is the ratio of perfusion and clearance rates, reflecting the amount of [^11^C]-acetate retention within the cell after clearance. In this study, the V_T_ value significantly differed between normal and cancerous prostate regions (p<0.01). From this small study group, it is possible to hypothesize that V_T_ might be able to differentiate cancerous region from normal prostate. Like K_1_, the V_T_ value is also influenced by partial volume effect. V_T_ was the kinetic parameter with the closest correlation to SUV from static imaging (r=0.8 for late SUV), confirming that what is measured in clinical whole-body PET/CT images can be used as an estimate of the anabolic metabolism of the tumor at the time of scanning. In addition, the retention of [^11^C]-acetate in the cells in vitro was high and in a good agreement with the data obtained from PET images of the studied patients in this study (Figure 2, 4). There was a significant difference (p=0.001) between the retention rates for DU145 and PC3 cell lines indicating that PC3 had a 2 times faster anabolic metabolism than DU145.

The investigation of the correlation of clinical parameters with SUV values and kinetic parameters provided additional support for the feasibility of PCa aggressiveness measurement using [^11^C]-acetate PET/CT. Correlation of PSA with early SUV (r=0.50, p=0.02) and K_1_ (r=0.48, p=0.03) suggests that cancer differentiation from normal tissue can be accomplished by [^11^C]-acetate PET/CT kinetic parameters. Similarly, correlation of both early and late SUVs with V_T_ (r>0.76, p<0.001) and K_1_ (r>0.64, p<0.005) signifies the use of SUV as a marker of prostate cancer. However, no significant correlation was noticed between Gleason score and PET parameters.

Previously, Tolbod et al. 41 non-invasively quantified tumor blood flow (TBF) in PCa using [^15^O]-water and concluded that [^15^O]-water PET can accurately measures TBF and be useful for evaluation of tumor aggressiveness in PCa. Further, a pilot study 42 was conducted to measure and quantify TBF in PCa using [^15^O]-water and [^82^Rb] PET/CT illustrated not only a good correlation of TBF for both tracers but also a significantly increased uptake of [^82^Rb] associated with PCa aggressiveness. These studies quantified increased blood flow in the cancerous region of prostate compared to normal and is also shown in this study (K_1_) using [^11^C]-acetate. Further, increased metabolic demand of cancerous region to support tumor growth reflected with increased [^11^C]-acetate retention (V_T_).

The results of the kinetic studies in vitro and in vivo indicate that the biological pathway of [^11^C]-acetate in prostate cancer cell was dominated by the uptake and retention most probably indicating the involvement of [^11^C]-acetate in lipid synthesis. As mentioned above the lipid synthesis extent is an indication of prostate cancer aggressiveness. The [^11^C]-acetate uptake and retention pattern in the cell experiments, and the correlation of the K_1_ and V_T_ parameters with SUVs in the clinical study clearly indicate that [^11^C]-acetate/PET has the potential for the estimation of the disease progression measuring the lipid synthesis extension.

Further improvement of the suggested methodology would require: inclusion of high risk aggressive prostate cancer patients to confirm the results attained from this study; introduction of partial volume correction for higher quantification accuracy; and histopathological documentation of a larger patient cohort.

## Conclusion

The single-tissue compartment model enabled generation of parametric images of [^11^C]-acetate kinetics for the visualization of cancer biology. The extraction rate (K_1_) and distribution volume (V_T_) of [^11^C]-acetate were considerably higher in low-to-intermediate risk prostate cancer as compared to normal prostate tissue in patients. These results were confirmed in vitro wherein faster growing PC3 cells showed elevated values of K_1_ and V_T_ as compared to DU145 cells. Thus, [^11^C]-acetate/PET demonstrated potential for the measurement of PCa growth and aggressiveness.

## Figures and Tables

**Table 1:** Demographic data on the age, PSA level, Gleason sum and risk assessment of patient cohort. * indicates patients with post-surgical ROIs.

Patient No.	Age (years)	PSA	Gleason sum	Risk
1	65	9.2	6	low
2*	67	12	7	moderate
3	72	3.2	5	low
4	62	4.6	7	moderate
5	59	6.1	6	low
6*	66	3.8	6	low
7	69	10	7	moderate
8	72	12	6	moderate
9	75	13	6	moderate
10	65	5.6	6	low
11	63	11	7	moderate
12*	68	4	7	moderate
13*	57	11	6	moderate
14	60	7.8	6	low
15	62	17	5	low
16*	69	13	7	moderate
17	74	11	6	moderate
18*	64	6	6	low
19	62	6	5	low
20*	51	4.8	6	low
21	62	3.8	5	low

**Table 2:** Dynamic [^11^C]-acetate PET/CT SUV findings along with parametric values in the study population. The data is presented as mean ± SD (n=21).

Parameters	Normal prostate	Cancerous prostate	p-value
SUV early	1.99±0.65	2.99±0.69	<0.01
SUV late	2.08±0.70	3.04±0.73	<0.01
K_1_ (ml/cm^3^/min)	0.23±0.05	0.34±0.09	<0.01
k_2 (_min^-1^_)_	0.05±0.01	0.04±0.01	0.26
V_T_ (ml/cm^3^)	5.44±1.52	8.6±2.52	<0.01

**Table 3:** Correlation of parametric values and SUV along with spearman correlation and respective p-values.

Correlation comparison		Spearman ρ	p-value
Early SUV	V_T_	0.76	<0.001
Early SUV	K_1_	0.65	0.001
Early SUV	PSA	0.5	0.02
Late SUV	V_T_	0.8	<0.001
Late SUV	K_1_	0.64	0.001
K_1_	V_T_	0.9	<0.001

## Abbreviations

PCa: prostate cancer
PSA: prostate specific antigen
FA: fatty acid
FASN: fatty acid synthetase
AceCS: acetyl-CoA synthetase
CT: computed tomography
MRI: magnetic resonance imaging
MRS: magnetic resonance spectroscopy
PET: positron emission tomography
SUV: standardized uptake value
TAC: time activity curve
OSEM: ordered subsets expectation maximization
VOI: volume of interest
ROI: region of interest
CPS: counts per second
